# Dynamic adaptation process to implement an evidence-based child maltreatment intervention

**DOI:** 10.1186/1748-5908-7-32

**Published:** 2012-04-18

**Authors:** Gregory A Aarons, Amy E Green, Lawrence A Palinkas, Shannon Self-Brown, Daniel J Whitaker, John R Lutzker, Jane F Silovsky, Debra B Hecht, Mark J Chaffin

**Affiliations:** 1 Department of Psychiatry, University of California, La Jolla, San Diego, CA, USA; 2 Child and Adolescent Services Research Center, Rady Children’s Hospital, San Diego, CA, USA; 3 School of Social Work, University of Southern California, Los Angeles, CA, USA; 4 Center for Healthy Development, Georgia State University, Institute of Public Health, Atlanta, GA, USA; 5 Department of Pediatrics, University of Oklahoma Health Sciences Center, Oklahoma, OK, USA

**Keywords:** Adaptation, Evidence-based practice, Implementation, Fidelity, Child maltreatment, Public sector

## Abstract

**Background:**

Adaptations are often made to evidence-based practices (EBPs) by systems, organizations, and/or service providers in the implementation process. The degree to which core elements of an EBP can be maintained while allowing for local adaptation is unclear. In addition, adaptations may also be needed at the system, policy, or organizational levels to facilitate EBP implementation and sustainment. This paper describes a study of the feasibility and acceptability of an implementation approach, the Dynamic Adaptation Process (DAP), designed to allow for EBP adaptation and system and organizational adaptations in a planned and considered, rather than *ad hoc*, way. The DAP involves identifying core elements and adaptable characteristics of an EBP, then supporting implementation with specific training on allowable adaptations to the model, fidelity monitoring and support, and identifying the need for and solutions to system and organizational adaptations. In addition, this study addresses a secondary concern, that of improving EBP model fidelity assessment and feedback in real-world settings.

**Methods:**

This project examines the feasibility, acceptability, and utility of the DAP; tests the degree to which fidelity can be maintained using the DAP compared to implementation as usual (IAU); and examines the feasibility of using automated phone or internet-enabled, computer-based technology to assess intervention fidelity and client satisfaction. The study design incorporates mixed methods in order to describe processes and factors associated with variations in both how the DAP itself is implemented and how the DAP impacts fidelity, drift, and adaptation. The DAP model is to be examined by assigning six regions in California (USA) to either the DAP (n = 3) or IAU (n = 3) to implement an EBP to prevent child neglect.

**Discussion:**

The DAP represents a data-informed, collaborative, multiple stakeholder approach to maintain intervention fidelity during the implementation of EBPs in the field by providing support for intervention, system, and organizational adaptation and intervention fidelity to meet local needs. This study is designed to address the real-world implications of EBP implementation in public sector service systems and is relevant for national, state, and local service systems and organizations.

## Background

Despite empirical support for evidence-based practices (EBPs) [1,2], widespread implementation with sustainment has been difficult to achieve across a variety of contexts and interventions. Moving EBP technologies from development and research settings to the practice setting, with fidelity, involves far more than simply making efficacious practice models available [3,4].

One of the critical challenges in large-scale implementations of EBPs is the tension between adaptation (*i.e.*, flexibility) and attaining fidelity [5-8]. Fidelity typically refers to an assessment of therapist adherence and competence [9]. *Adherence* refers to the extent that the techniques implemented in a session match the intentions of the model developers as well as to the more structural elements of fidelity, such as dosage of treatment and frequency of supervision [10]. *Competence* refers to the provider skills used to deliver the model, including responsiveness to the behaviors of a client and selection of appropriate intervention components [9]. For the purposes of this paper, we define adaptation as “to make fit (as for a specific or new use or situation) often by modification” [11]. This definition leaves room for adaptation of both an EBP and of the context into which the EBP is to be implemented.

The interplay of characteristics of an intervention with service system and organizational characteristics (system, organization, provider, and client levels) can be complex [12]. Such complexity may impact the need to adapt interventions to the service context and to adapt aspects of outer context (*i.e.*, service system) and inner context (*i.e.*, organization) to effectively implement EBPs [13,14]. This is akin to the concepts of accommodation and assimilation found in developmental cognitive psychology [15]. For example, a service system may accommodate an EBP by changing funding and contracting in order to support the intervention. In contrast, if funding and contracting are already in place, then assimilation of the EBP into the service system can be made with little systemic or organizational adaptation.

The need for treatment or intervention adaptation has been highlighted in a wide range of EBPs, including for child maltreatment interventions [16,17], substance abuse treatment [18], child anxiety interventions [5], HIV treatment [19], school-based social competence interventions [20], psychological treatments for a variety of disorders [21], anorexia nervosa treatment [22], and health risk prevention programs [23-25]. In spite of this need, there is often the expectation that EBPs be delivered with strict adherence to standards that were developed for efficacy trials. Strict adherence may be at odds with broader implementation of EBPs in real-world practice settings, thus, raising concern about the balance between delivering EBPs with fidelity and making adaptations believed to be necessary for usual care contexts. This “adaptation-fidelity” tension necessitates a better understanding of how to facilitate delivery of EBPs with appropriate adherence and competence, while allowing for adaptations that do not interfere with core elements (*i.e.*, intervention components believed to be necessary to attain intervention effects). Models of planned adaptation are now being developed but are only beginning to be tested [26], and recent initiatives have begun to support empirical study of implementation processes and outcomes.

Intervention adaptation at its best is a cautious process designed to allow an EBP to be delivered faithfully in situations where it otherwise might not fit. Some typical examples include reordering components, forestalling or delaying certain components, de-emphasis and emphasis, augmentation (adding materials or interventions) of components, and language and cultural adaptations [17,27]. Other examples include evolutionary improvements to a model. In both senses, adaptation is a positive process.

In contrast, *drift* is a misapplication or mistaken application of the model, often involving either technical error, abandonment of core and requisite components, or introduction of counterproductive elements. Drift occurs easily in field implementations, especially among organizations and practitioners that have not yet achieved full competency or integration of a new model and are not in consultation with model experts [28]. Drift is often found to result in loss of downstream client benefits [29,30].

**Figure 1:**
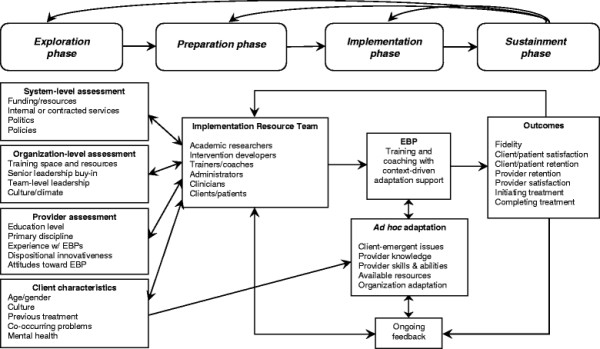
Conceptual model guiding the Dynamic Adaptation Process to support effective evidence-based practice implementation representing the four phases (Exploration, Preparation, Implementation, Sustainment) of the EPIS implementation conceptual model [13].

EBP implementation with fidelity may also require adaptations to service system and organization policies, processes, and structure as the social and organizational context can influence the process of implementation [13,31]. For example, in the outer context, communities may need to develop alignments among stakeholders or change funding or contracting in order to successfully implement an EBP [32,33]. There may also be a need to address inner context issues, such as staff retention, organizational culture and climate, or organizational structure (*e.g.*, supervision) [13,34]. For example, intervention may be needed to improve leadership or implementation climate at one or more levels within provider organizations. Because there has been limited research on models that allow for intervention and contextual adaptation while maintaining both structural fidelity [10] and fidelity to the core elements of an EBP, we proposed a model based on current literature and our own research and experience in multiple EBP implementation studies. As shown in Figure 1, the Dynamic Adaptation Process (DAP) provides a four-phased process for implementing an EBP that takes into account the multilevel context of services delivery, engages multiple stakeholders, and provides appropriate expertise and feedback during implementation to guide, monitor, and address system, organization, and model adaptations while maintaining fidelity to the core elements of an EBP.

A second, related concern addressed by this study is the development of practical and cost-effective fidelity assessment methods. Such methods are needed in order to move EBPs into usual care settings and monitor variations in fidelity to be utilized in the DAP. Behavioral and psychosocial EBPs implemented in research contexts often use *in vivo* observations or coding of video- or audio-recorded sessions to monitor fidelity, and such approaches are expensive and time consuming [35]. Some interventions have successfully used client report to assess fidelity during research studies but this approach may be vulnerable to demand characteristics that may affect fidelity ratings if administered by providers [36]. More efficient approaches are needed as fidelity assessment can be labor intensive, requiring additional personnel to contact clients and conduct fidelity assessment data collection to reduce demand characteristics [37]. The use of technological innovations to collect fidelity data from clients may present a less time-intensive method of collecting these data while also minimizing demand characteristics.

The current study contributes to implementation science by addressing the issue of adaptation in a large, diverse state context by experimentally manipulating the implementation approach. The study context consists of multiple regions in the state of California implementing SafeCare© (SC), a behavioral and psychosocial EBP developed to prevent child neglect [38]. The SC model, which grew out of the behavior analysis field, is manualized and highly structured and uses classic behavioral intervention techniques (*e.g.*, ongoing measurement of observable behaviors, skill modeling, direct skill practice with feedback, training skills to criterion) [39]. Behavioral theory conceptualizes child neglect in terms of skill deficits, particularly those skills that are most proximal to neglect and that form the objective basis for the family’s involvement in the child welfare system—such as failing to provide adequate nutrition, healthcare, cleanliness and a safe home environment; parental disengagement; low levels of parental supervision; and inappropriate parenting or child-management. SC is comprised of three modules addressing these issues: infant and child health, home safety and cleanliness, and parent–child (or parent-infant) interactions. An additional component of SC involves the use of observations and coaching from model experts. Finally, the United States National SafeCare Training and Research Center (NSTRC) has developed a “train-the-trainer” model, in which selected providers can eventually be trained and certified as SC coaches and trainers in order to sustain and expand local implementation [40].

In applying the DAP model to SC implementation, the investigative team works along with child-welfare system directors and staff, program leaders, clinicians, and model developers to use the DAP to guide and provide appropriate adaptation of the EBP and the service context. Up to 12 counties across six regions will be randomly assigned to be trained in SC implementation as usual (IAU) versus the DAP approach. Although process and implementation evaluations have traditionally relied upon the use of qualitative methods, [41] the current study goes beyond this to apply mixed quantitative-qualitative methods [42] to compare two different implementation strategies.

The specific aims of this study are as follows:

· Aim 1: Use the DAP to modify SC training and ongoing SC coaching to support adaptation of SC in practice.

· Aim 2: Use qualitative methods to examine the process, feasibility, acceptability, and utility of the DAP.

· Aim 3: Test whether DAP implementation results in (a) fidelity to SC core elements equal to IAU, (b) greater provider engagement with the implementation, or (c) improved client satisfaction compared to IAU.

· Aim 4: Examine organizational and provider factors likely to impact adaptation and implementation outcomes.

· Aim 5: Test the utility of technological solutions for collecting client fidelity assessment and satisfaction data.

## Methods/design

### Study context

The context for this study includes multiple counties in the state of California, USA. As such, SC implementation will occur at the county level, since it is at this level that child-welfare and home-visitation service systems in California are administered. Each year, two counties (or a consortia of counties) blocked on similar characteristics (*e.g.*, urban/rural) are to be selected from a competitive process that is part of outside funding from the US Administration for Children and Families to participate in an Evidence-Based Home Visitation project. In each of three years, two counties (or a consortia of counties) are assigned by a coin flip to either the DAP or to IAU. DAP and IAU conditions are assigned different certified trainers to avoid cross-contamination. Separate pairs of applicants are selected each year over the three-year project to create a sample sufficient to test the utility of the DAP and learn about its process and impacts. In the DAP condition, adaptations are explicit and done in a planned way by the Implementation Resource Team (IRT), in conjunction with service providers and coaches, in order to preserve fidelity to core components. In the IAU condition, adaptations are likely to be more *ad hoc* and idiosyncratic. The IAU condition has a less rigorous and more informal assessment process in the Preparation phase, relative to the DAP condition. Still, most SC implementations include technical assistance and coaching following training. As such, adaptations, while *ad hoc*, are often done with guidance from the model developers, albeit in a less formal or systematic way than that proposed here.

### Aim 1: Use the DAP to modify SC training and ongoing consultation to support adaptation of SC in practice

#### The dynamic adaptation process

As shown in Figure 1, the DAP involves the four phases of the EPIS implementation conceptual model (Exploration, Preparation, Implementation, and Sustainment) [13] that, while generally sequential, allow for feedback to earlier phases. This process is continuously iterative, in that ongoing experience can inform continued adaptation as needed. Other core features of the DAP are collaboration of an IRT comprised of multiple stakeholders, providing client feedback and the data based on client surveys to coaches, and making adaptation an explicit part of the implementation process. The IRT consists of experts in SC and implementation science, as well as members of the county and organizations involved in the local implementation. The IRT meets monthly via a conference call or in person to examine adaptation needs and fidelity/satisfaction data and guide the implementation with adaptation support. Each phase of the DAP is described below.

#### Exploration phase

Consistent with the need to consider the multilevel nature of the service context [43-46], this phase involves a multilevel assessment of system, organization, provider, and client characteristics. A continuous information feedback loop is created such that information gathered during the assessments in this phase are used by the IRT to make adjustments to the way that SC is trained and delivered so that it can be implemented effectively in each local context while retaining fidelity to the SC model. During the Preparation phase, the following levels are assessed through a semi-structured interview with stakeholders at the system, organization, and provider levels in the DAP condition.

*System-level assessment* involves working with county child-welfare system and agency leaders to determine if the prerequisite conditions exist that will facilitate the implementation and sustained use of SC.

*Organization-level assessment* involves both practical concerns (*e.g.*, training space and resources, proximity to clients, transportation availability) and organizational factors associated with successful agency operation and implementation readiness (senior leadership, team-level leadership, organizational culture and climate). These latter constructs are associated with staff readiness to adopt EBPs [44,47,48], as well as client outcomes [49] and implementation effectiveness [50]. For system and organizational assessment, we conduct key informant interviews with county administrators, agency directors, and providers addressing both implementation readiness and adaptation needs.

*Provider-level assessment* involves a staff survey that assesses individual factors, including staff demographics, experience with home-visitation services, personal dispositional innovativeness (willingness and desire to experiment with new procedures, new tasks, or new ways of helping clients), work attitudes, and attitudes toward EBPs, as well as organizational factors such as organizational culture, climate, and leadership (factors shown to be associated with EBP implementation). For example, in terms of demographics, higher educational attainment is associated with more positive attitudes toward adopting EBPs [44]. Knowledge of provider factors can help trainers understand provider attitudes and perspectives and tailor training accordingly. In addition, understanding personal dispositional innovativeness will help trainers tailor training to the level of flexibility or rigidity within a team.

To assess *client characteristics*, we obtain and assess surveillance data for the catchment area based on county reports from local authorities (*e.g.*, child-welfare system, substance abuse treatment system, mental health system), agency reports based on local expertise, and provider reports of their experience with representative clients. The dynamic nature of the DAP allows us to make adjustments based on feedback from ongoing home-visitor reports, client data, and information about the need for *ad hoc* adaptations fed back to the ongoing coaches and IRT. Assessing individual clients in the Preparation phase is not practical as we do not know who will be coming into the service system. Once clients are engaged in services, *ad hoc* adaptations may be made in the Implementation phase.

#### Preparation phase

This phase involves making information gathered in the Exploration phase available to the entire IRT. The IRT examines exploration phase results, descriptions of service contexts, data reports, and other materials pertinent to adaptation in the proposed service context to determine what adaptations may be needed in the service context and how such adaptations are to be accomplished.

#### Implementation phase

Based on the outcome of the Adoption Decision/Preparation phase, training with adaptation support begins in the Implementation phase. In contrast to the IAU condition in which the curriculum is set, the DAP training supports changes deemed necessary by the IRT. One prominent difference between IAU and DAP conditions is the explicit inclusion and discussion of adaptation during provider training, including why one might adapt, what one might adapt, what one might not adapt, when to seek guidance on adaptation, and how to use the ongoing coaches and IRT for tailoring SC. In addition to intervention adaptation, the need for adaptation at the system and/organizational levels is also an ongoing target for change. In addition, the research team in conjunction with intervention developers will refine assessment of fidelity. Departures from fidelity to core elements will be considered drift.

#### Sustainment phase

The Sustainment phase involves ongoing use of client and system data to provide feedback to the coaches and the IRT who can use that information to better understand home-visitor fidelity, client satisfaction with services, and client satisfaction with SC. This information is collected in both the DAP and IAU conditions but is only fed back to DAP coaches on a monthly basis. One of the main benefits of this information is that DAP coaches will have access to data from all of the SC clients rather than only the one or two per month who are observed during *in vivo* coaching sessions. Client satisfaction data are also used in the DAP condition to monitor the perception of the relationship between the client and home visitor to help support coaching around maximizing client engagement in services.

### Aim 2: Use qualitative methods to examine the process, feasibility, acceptability, and utility of the dynamic adaptation process

#### Participants

This qualitative portion of the study will involve recruiting a total of 30 home visitors and all team leaders/clinical supervisors (n = 6; one from each team) from each agency implementing SC. All county child-welfare directors and all agency directors (*i.e.*, subcontractors or program leaders) will be recruited to participate because their perspective on the DAP approach and implementation of SC is essential to understanding the process of implementing an EBP. Inclusion of representatives across the state of California may reveal different needs and concerns related to unique regional issues (urbanicity, ethnic variation, culture, socioeconomic status, availability of resources and services, politics, policies) that play into delivering SC with fidelity and the utility of the DAP approach. Using a maximum-variation sampling procedure, purposeful (*i.e.*, not random) recruitment of up to 30 provider staff will proceed until it is determined that sufficient saturation (*i.e.*, collection of the same information from more than one informant) of responses to the interview protocol was obtained through an iterative process of data collection and analysis.

#### Data collection and analysis

The qualitative analysis of the DAP will include three interrelated methods of collecting data: (1) *in vivo* observation of the IRT, home visitors, trainings, and coaching, with detailed field notes prepared by the ethnographer; (2) extended semi-structured interviews with IRT members, home visitors, and child-welfare directors using an interview guide designed to elicit information on knowledge, attitudes, and behavior related to the use of the DAP and the implementation process; and (3) focus groups with home visitors and the IRT to elicit comments on the implementation process and the utility of the DAP conceptual model. All three forms of qualitative data collection will be conducted by an ethnographer under the supervision of a medical anthropologist. Accuracy of information obtained through the different data collection methods will be assessed through a process of triangulation in which accounts of specific events and behaviors obtained from observation field notes, interviews, and focus groups are compared with one another to determine if they converge in providing the same or similar answers to the same questions. The empirical material contained in the field notes, interviews, and focus group sessions will be independently coded by the project investigators to condense the data into analyzable units and analyzed with the computer program QSR NVivo [51]. A concurrent mixed-methods approach will utilize triangulation and examination of convergence, complementarity, and expansion to integrate quantitative and qualitative data and results [42].

### Aim 3: Test whether DAP implementation results in fidelity to SC core elements equal to implementation as usual as well as greater client satisfaction with the implementation

#### Participants

The primary direct participants are agency staff who will be delivering SC (n = 72). Because the main quantitative aims of the study involve provider fidelity and factors related to fidelity, agency staff will be the main subjects of study. Clients also will be involved as participants but to a far lesser extent, via de-identified administrative data, and as a source for fidelity ratings of provider staff. All clients receiving SC from a provider enrolled in the study will be eligible for inclusion. Clients will be selected by the agencies and child welfare to receive SC based on two criteria: (a) a child-welfare referral or concern about child neglect and (b) at least one child in the family under age eight who is considered at risk for neglect. Clients will be excluded only if they are unable to comprehend or provide data. The minimum number of eligible client participants is estimated to be 720.

#### Measures

##### Fidelity

We will use two sources of fidelity data: direct observation methods and client report. One has the advantage of expertise and objectivity, and the other has the advantage of high frequency availability and relevance to client perspectives. SC sessions will be observed and will be coded by coaches for each observed session using the SC Fidelity Checklist Tool (two to four sessions monthly). For client report, we are using a parallel version of the SC Fidelity Checklist Tool. The multisource measurement occasions will provide the opportunity for detailed comparisons between fidelity information gathered from clients and from observers. The more detailed client-report data gathered on a weekly basis will provide opportunities to examine patterns of change in fidelity over time and provide data to the coach and adaptation team.

##### Satisfaction

Client satisfaction with SC is assessed using the model developers’ client satisfaction scales that assess satisfaction with each of the SC modules.

##### Client engagement in services

Client retention and recidivism data will also be compared between the IAU and DAP using data obtained from county child welfare databases.

##### Data analysis

Equivalence testing [52-54] will be used to evaluate whether DAP implementation results in fidelity to SC core elements equal to IAU. We expect that home visitors in the DAP teams will attain approximately 88% treatment fidelity (100% is perfect fidelity). A value of ± Δ_*B*_ = 12% was set as a bound that would indicate non-substantive group differences (*i.e.*, group equivalence) in treatment fidelity between the DAP and IAU groups. Therefore, equivalence exists if the IAU group has a fidelity value between 76% and 100% in the population. Using the bounds specified above, along with a hypothesized difference between population group means of zero, and a standard deviation of 15 in each group, the proposed sample size (n = 72) would yield power of .91 to reject the null hypothesis of mean differences in favor of the alternative of group equivalence. In order to evaluate differences between DAP and IAU on client satisfaction, a hierarchical linear regression model addressing clustering at the team level that includes a dummy-coded grouping variable and relevant covariates (*e.g.*, organizational culture/climate) will be evaluated. The effect posited for the difference between the DAP and IAU groups was set to medium, *d* = .50, which, converted to a correlation [55], yields an *r* value of .24. For the client sample size of 720, the analyses would yield power greater than .99 for test of the group effect. Finally, client retention rates and re-report rates across conditions will be assessed as a time-to-event outcome using survival analysis techniques. Analysis time will be measured in days from intake through program dropout or program completion (right censored data).

### Aim 4: Examine organizational and provider factors likely to impact adaptation and implementation outcomes

#### Participants

To examine aim 4, agency staff (n = 72) delivering SC are administered biannual web surveys regarding individual and organizational level factors hypothesized to impact implementation outcomes.

#### Measures

*Work attitudes* are measured across all providers nested within agencies using measures of job satisfaction and organizational commitment from the Organizational Social Context Scale (OSC) [56].

*Turnover intentions* are assessed using five items derived from organization studies and adapted for use in human service agencies [57].

*Personal dispositional innovativeness* is assessed using the Adaptability, Change Catalyst, and Conscientiousness scales of the Emotional Competence Inventory (ECI) [58].

*Provider satisfaction with SC* is assessed with a 10-item, two-factor scale of Provider Knowledge (four items) and Perceived Value (six items) of SC.

*Attitudes towards adopting EBP* is assessed using the Evidence-Based Practice Attitude Scale (EBPAS), which examines four dimensions of attitudes toward adoption of EBPs: (1) intuitive *appeal* of EBP, (2) likelihood of adopting EBP given *requirements* to do so, (3) *openness* to new practices, and (4) perceived *divergence* between research-based/academically developed interventions and current practice.

#### Data analysis

In order to test differences between DAP and IAU on work satisfaction and turnover intentions, separate hierarchical linear regression models addressing clustering at the team level, with a dummy-coded grouping variable and relevant covariates, will be evaluated. Exploratory analyses will involve examining the Pearson product moment correlations among personal dispositional innovativeness, provider satisfaction with SC, and attitudes towards adopting EBP, with a series of the correlation coefficients both within and across conditions (DAP vs. IAU). The direction and magnitude of these correlations, as well as their associated confidence intervals, will be used to judge effect size magnitude.

### Aim 5: Test the utility of technological solutions for collecting client fidelity assessment and satisfaction data

#### Participants

The primary direct participants are agency staff who will be delivering SC (n = 72). All clients receiving SC from a provider enrolled in the study will be eligible for inclusion. The minimum number of eligible client participants is estimated to be 720.

### Measures

#### Fidelity

Fidelity data will be collected via direct observation methods or client phone or computer report using the SC Fidelity Checklist Tool as described under aim 3. Client data regarding their provider visits are collected on a weekly basis and direct observation data of providers are collected two to four times per month. At the end of each SC visit, home visitors provide clients with either an auditory survey administered using automated telephone technology or an online form administered through a wireless-enabled netbook. Use of these technological approaches will replace the need for the home visitor to prepare a paper-based fidelity form for each home visit, reducing the fidelity-monitoring burden for the home visitor and client and decreasing demand characteristics of having the home visitor give clients the fidelity measure that is to be returned to the home visitor. The multisource measurement occasions will provide the opportunity for detailed comparisons between fidelity information gathered from clients and observers.

#### Data analysis

A weighted kappa statistic [59] will be used to evaluate level of agreement between fidelity ratings using phone or computer technology and observer matched to the same sessions.

## Discussion

The degree to which core elements of EBPs can be maintained while allowing for local adaptation is unclear, and concern is reflected in prevention and intervention literatures. In this project, we develop and evaluate the DAP, an implementation approach that uses data collection and feedback processes to prepare and support systems, organizations, and service staff to inform appropriate adaptations to both the EBP and the service context. The DAP involves identifying and distinguishing core elements and adaptable characteristics of an EBP, then supporting implementation of the adapted model. It also identifies system and organizational characteristics requiring adaptation for effective implementation. By using a mixed-method approach to examine each of the specific aims outlined in this study protocol, we aim to advance implementation science by addressing the tension between adaptation and fidelity and examine mechanisms and methods to improve fidelity assessment. The work described here should serve to advance the relatively nascent science of adaptation during implementation. If successful, the approach described here may be of value in other efforts to scale-up EBPs in order to improve public health outcomes.

The DAP model presented here also builds synergistically with our work on implementation in public sector service systems and organizations. By combining our phased, multilevel conceptual model, we have developed a general model of implementation. As shown in Figure 1, our strategy—although yet to be tested—allows for preassessment, problem solving, and outcomes feedback through the four EPIS conceptual model implementation phases of Exploration, Preparation, Implementation, and Sustainment [13]. Our approach also brings together relevant stakeholders to maximize the likelihood of effective EBP implementation and sustainment.

## Competing interests

GAA is an Associate Editor of *Implementation Science*; all decisions on this paper were made by another editor. The authors declare that they have no other competing interests.

## Authors’ contributions

GAA is the principal investigator for the described study. GAA conceptualized and designed the study, drafted the manuscript, and approved the final version. AEG contributed to the conceptualization and design of the study, drafted the manuscript, and approved the final version. LAP, SSB, DJW, JRL, JFS, DBH, and MJC contributed to the conceptualization and design of the study, revised the manuscript, and approved the final version.
